# Linagliptin loaded chitosan nanoparticles for the efficient therapy of renal fibrosis

**DOI:** 10.3389/fphar.2026.1773015

**Published:** 2026-03-19

**Authors:** Zhixiang Wu, Yanjuan He, Hao Deng, Yue Tan, Wangzi Gao, Jiaxin Yin, Ziqin Zeng, Jing Sun, Lijuan Zhang, Yue Li, Shentang Li

**Affiliations:** 1 Department of Pediatrics, The Third Xiangya Hospital, Central South University, Changsha, China; 2 Department of Pediatrics, The Fourth Hospital of Changsha, Changsha Hospital of Hunan Normal University, Changsha, China; 3 Hunan Normal University School of Medicine, Changsha, China

**Keywords:** anti-renal fibrosis, biocompatibility, chitosan nanoparticles, chronickidney disease, dipeptidyl peptidase-4 inhibitor, drug delivery system, linagliptin, nano-delivery carrier

## Abstract

Chronic kidney disease (CKD), whose prevalence is rising substantially, has become a major global health challenge. In this study, we developed linagliptin-loaded chitosan nanoparticles (HCS-LGP NPs) for the treatment of renal fibrosis. The synthesized nanoparticles exhibited a uniform particle size of 178.8 ± 4.4 nm and a zeta potential of −29.9 ± 2.5 mV, along with acid-responsive drug release properties. *In vitro*, the HCS-LGP NPs showed efficient cellular uptake in TGF-β1-induced HK-2 cells and significantly inhibited cell proliferation by downregulating the expression of TGF-β1 and Collagen I. *In vivo* studies demonstrated effective renal accumulation of HCS-LGP NPs in rats with renal fibrosis. Treatment with HCS-LGP NPs significantly reduced serum creatinine, blood urea nitrogen, and the protein levels of TGF-β1 and Collagen I. Notably, the administration of HCS-LGP NPs promoted marked recovery from renal injury and markedly reduced collagen fiber deposition in rats with renal fibrosis. Histopathological analysis confirmed excellent biocompatibility, with no observable damage to the heart, liver, spleen, or lungs. These findings indicate that HCS-LGP NPs hold great potential as a targeted therapy for renal fibrosis, offering enhanced efficacy and a favorable safety profile.

## 1 Introduction

Chronic kidney disease (CKD) is a progressive condition marked by persistent structural deterioration and functional decline of renal tissue, posing a substantial global health burden with a steadily increasing prevalence (Chen et al., 2019). Epidemiological studies indicate that China carries the highest CKD burden in Asia, accounting for 36.80% of adult cases in 2022 (Liyanage et al., 2022). As a result, developing effective prevention and treatment strategies has become an urgent research priority. The primary etiological factors of CKD include diabetes mellitus and hypertension (Mccarty, 2006; Tuttle et al., 2022), while secondary causes encompass infectious glomerulonephritis, renal vasculitis, ureteral obstruction, genetic disorders, autoimmune diseases, nephrotoxic medications, among others (Baudoux et al., 2022; Li et al., 2023; López-Novoa et al., 2010; Satoskar et al., 2020; Moiseev et al., 2017; Nørregaard et al., 2023; Kato and Natarajan, 2019; Rodrigues et al., 2017). A range of progressive renal pathologies can lead to renal fibrosis, which is characterized by excessive extracellular matrix accumulation, impaired matrix degradation, altered receptor signaling, fibroblast activation and myofibroblast differentiation, epithelial-mesenchymal transition, immune cell infiltration, and dysregulated apoptosis (Huang et al., 2023).

Linagliptin (LGP), a specific dipeptidyl peptidase-4 (DPP-4) inhibitor, is clinically used for the treatment of type II diabetes. Its primary mechanism involves inhibiting the degradation of glucagon-like peptide-1 (GLP-1) and glucose-dependent insulinotropic peptide (GIP) by DPP-4, thereby elevating levels of active incretin hormones. This leads to glucose-dependent insulin secretion, reduced circulating glucagon, and consequent blood glucose reduction (Hou et al., 2006). Beyond glycemic control, DPP-4 inhibitors exhibit pleiotropic biological effects due to the involvement of DPP-4 in cleaving various natural substrates such as stromal-derived factor-1 alpha (SDF-1α), brain natriuretic peptide (BNP), and neuropeptide Y (NPY), among others (Hsu et al., 2014; Heerspink et al., 2020). Notably, accumulating evidence indicates that LGP offers significant renoprotective benefits, including attenuation of proteinuria, glomerulosclerosis, and tubulointerstitial fibrosis (Wheeler et al., 2021; Gerstein et al., 2021; Mann et al., 2017; Heerspink et al., 2019).

The GLP-1/GLP1R pathway appears to play only a secondary role in the renal benefits conferred by LGP. Studies conducted in GLP1R-knockout mice and 5/6 nephrectomized (5/6Nx) wild-type mice demonstrated that LGP downregulates Y-box binding protein 1 (YB-1) in the kidney independently of GLP1R. This effect was mediated through the upregulation of heterogeneous ribonucleoprotein A1 and thymosin-β4, resulting in the subsequent downregulation of both YB-1 and transforming growth factor-β1 (TGF-β1), thereby mitigating renal impairment induced by 5/6Nx (Torres et al., 2017). Furthermore, LGP was shown to attenuate methylglyoxal (MGO)-induced peritoneal fibrosis in mice, inhibit inflammatory cell infiltration into peritoneal tissues, and reduce TGF-β1 levels in peritoneal fluid (Bakris et al., 2020). Additionally, LGP normalized endothelial DPP-4 levels and directly inhibited the DPP-4/ITGb1 interaction, leading to suppression of pathological TGF-β1 signaling and VEGF responses (Del Prato, 2011). Collectively, these findings indicate that the antifibrotic effects of LGP involve the inhibition of aberrant TGF-β1 expression via both direct and indirect mechanisms.

To date, oral administration remains the most common route of drug delivery in both clinical practice and laboratory research. However, orally administered drugs often suffer from limited bioavailability, as their dissolution rate and membrane permeability are constrained by intrinsic properties such as solubility and molecular weight (Hocher et al., 2012; Scheen, 2018). Furthermore, after ingestion, active pharmaceutical ingredients undergo extensive physicochemical changes throughout the oral cavity and gastrointestinal tract, including enzymatic degradation due to gastric acidity and microbial metabolism in the gut (Kanasaki, 2018). Notably, LGP—classified as a Biopharmaceutics Classification System (BCS) class II drug and a substrate of P-glycoprotein (P-gp)—exhibits several pharmacokinetic challenges. These include P-gp-mediated efflux, significant first-pass metabolism, poor permeability, and widespread systemic distribution, all contributing to its low oral bioavailability (29.5%) (Karimifar et al., 2023). In diabetic nephropathy models, the combination of telmisartan and LGP significantly reduced urinary microalbumin, whereas LGP monotherapy showed no significant effect (Ott et al., 2016). A phase 3b randomized, controlled, double-blind clinical trial involving patients with early type II diabetic nephropathy demonstrated blood glucose reduction after 24 weeks of LGP treatment. Although a trend toward reduced urinary microalbumin was observed, it did not reach statistical significance (Coppolino et al., 2018). These findings suggest that LGP holds promise as a therapeutic agent for attenuating renal fibrosis, lowering urinary microalbumin, and preserving renal function. Nonetheless, strategies to enhance its efficacy are warranted.

**FIGURE 1:**
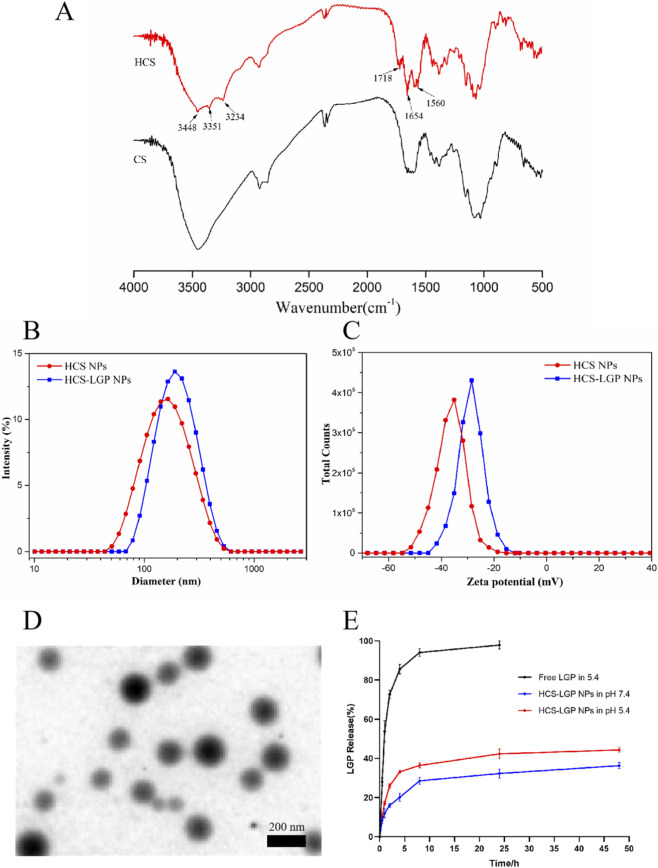
**(A)** FT-IR spectra of CS and HCS polymers. Particle size **(B)** and Zeta potential distribution **(C)** of HCS NPs and HCS-LGP NPs. **(D)** TEM image of HCS-LGP NPs. **(E)** Drug release profiles of Free LGP and HCS-LGP NPs in PBS at pH 5.4 or pH 7.4 for 48 h.

Nano drug delivery systems offer promising strategies to enhance drug solubility, modulate *in vivo* distribution, and improve targeting capability, thereby increasing therapeutic efficacy while minimizing adverse effects (Hasan et al., 2019; Nagai et al., 2016). Chitosan is widely utilized as a nanocarrier for antitumor and antibacterial agents owing to its excellent biocompatibility and ease of chemical modification (Zeisberg and ZeisberG, 2015). Of particular significance, low molecular weight chitosan (LMWC) demonstrates renal-specific uptake. This targeting behavior may originate from the aminoglycoside-like structure of LMWC, which mimics ligands for the Megalin receptor—highly expressed on the apical membrane of renal proximal tubular epithelial cells (Selby-Pham et al., 2017; Veber et al., 2002; Cheng and Wong, 2020; Shah et al., 2021; Alter et al., 2012). In this study, we synthesized an amphiphilic copolymer by conjugating sulfadimethoxypyrimidine (SDM) to LMWC via a succinic anhydride linker (Figure 1). Comprising a hydrophilic backbone and hydrophobic side chains, this copolymer self-assembles in aqueous solution into nanoparticles with a stable core–shell structure, driven by hydrophobic interactions. LGP was subsequently encapsulated within the hydrophobic core of these nanoparticles through physical entrapment.

## 2 Materials and methods

### 2.1 Materials

Chitosan (CS, MW 50,000), LGP (99%), 1-ethyl-(3-dimethylaminopropyl) carbodiimide hydrochloride (EDCI) were purchased from Shanghai Macklin Biochemical Technology Co., Ltd. SDM was obtained from Shanghai Eno Chemical Technology Co., Ltd. Succinic anhydride was purchased from Shanghai Aladdin Biochemical Technology Co., Ltd. Dialysis bags (MWCO 8000-14,000 and 3,500 Da), fluorescein isothiocyanate (FITC), adenine were obtained from Shanghai Yuanye Biotechnology Co., Ltd. Dimethyl sulfoxide (DMSO), succinic anhydride (SAA), and other materials were purchased from Sinopharm Chemical Reagent Co., Ltd.

### 2.2 Animals and cell cultures

HK-2 cells were was obtained from the American Type Culture Collection. The complete growth medium was DMEM (10% FBS, 1% penicillin/streptomycin). The cells were cultivated in an incubator (Thermo Scientific, 37 °C, 5% CO_2_).

The SD rats (5–6 weeks, 200–220 g) were purchased from Shanghai Laboratory Animal Center, Chinese Academy of Sciences. The models of renal fibrosis were set up via the administration of adenine (200 mg/kg, once a day for 4 weeks).

### 2.3 Synthesis of hydrophobically modified chitosan polymer (HCS)

Chitosan (CS, 0.5 g) was dissolved in 15 mL of acetic acid solution (5%, v/v) and then diluted with 20 mL of DMSO. To this solution, SAA was added at a molar ratio of CS to SAA of 1:4. The reaction mixture was stirred at 60 °C for 24 h to yield N-Succinyl chitosan (NCS). Subsequently, SDM and EDCI were introduced into the NCS solution at a molar ratio of CS to SDM to EDCI of 1:0.3:0.3. The reaction was carried out at 60 °C for another 24 h. After cooling to room temperature, the mixture was dialyzed against deionized water using a dialysis membrane (MWCO: 8,000–14,000 Da) for 24 h with frequent changes of water. Finally, the dialyzed product was freeze-dried to obtain hydrophobically modified chitosan (HCS).

### 2.4 Preparation of drug-loaded nanoparticles (HCS-LGP NPs)

HCS (50 mg) was dispersed in 50 mL of PBS (pH 9.18) to form solution A. Separately, 5 mg of LGP were dissolved in 10 mL of methanol (solution B). Solution B was then added dropwise into solution A under vigorous stirring. The mixture was stirred vigorously at 37 °C for 24 h. Subsequently, it was sonicated (200 W) in an ice bath for 5 min until a nearly transparent, light yellow solution was obtained. Finally, the solution was filtered through a 0.45 μm membrane and lyophilized to yield the HCS-LGP nanoparticles (HCS-LGP NPs). For the preparation of blank HCS NPs, an equivalent volume of methanol without LGP was used in place of solution B.

### 2.5 Characterization

The structure of HCS was characterized by FT-IR and ^1^H NMR spectroscopy. The morphology of both HCS-LGP NPs and HCS NPs was observed using transmission electron microscopy (TEM, HT7800) at 20 kV. The size and Zeta potential measurements were performed on a Malvern Zetasizer Nano-ZS instrument (Malvern Instruments, Malvern, United Kingdom) at 25 °C after appropriate dilution with deionized water. Reported values represent the average of three independent measurements.

The amount of LGP loaded in HCS-LGP NPs was quantified by UV spectrophotometry (Beckman DU800) at a wavelength of 300 nm. A predetermined calibration curve of LGP was used for concentration calculation.

$$\text{Drug loading }\left(\%\right)=\frac{\text{Weig}ht\;\;\text{of}\;\text{drugs}\;\;\text{in}\;\text{nanoparticles}}{\text{Weig}ht\;\;\text{of}\;\;\text{nanoparticles}}\times 100\%$$

### 2.6 *In vitro* drug release of HCS-LGP NPs

*In vitro* drug release from the nanoparticles was evaluated using a dialysis method. Specifically, HCS-LGP NPs or free LGP were dispersed in 10 mL of PBS buffer and introduced into a pre-hydrated dialysis bag (MWCO 3500 Da). The dialysis bag was then immersed in 200 mL of 0.1 M PBS (pH 7.4 or 5.4) and continuously agitated at 100 rpm in a shaker incubator at 37 °C. The concentration of the released drug was monitored by UV spectrophotometry at 300 nm.

### 2.7 Cellular uptake

FITC-labeled LGP was prepared by conjugating fluorescein isothiocyanate (FITC) to LGP via an amido bond, enabling its use as a fluorescent probe. Confocal imaging was performed using a Leica laser scanning confocal microscope. FITC was excited at 488 nm, and its emission was collected between 500 and 550 nm.

HK-2 cells were seeded and preincubated for 24 h at 37 °C under 5% CO_2_. Subsequently, the cells were incubated with FITC-labeled HCS nanoparticles for 8 h. In control experiments, HK-2 cells were treated with free FITC under identical conditions. Before imaging, all cells were washed twice with PBS buffer.

### 2.8 Cell viability

The cytotoxicity of HCS-LGP NPs was evaluated using the MTT assay. Briefly, HK-2 cells in the exponential growth phase were seeded in quintuplicate into a 96-well flat-bottomed microplate and incubated for 24 h in culture medium containing HCS-LGP NPs. After the incubation, 20 μL of MTT solution (5 mg/mL in PBS) was added to each well, and the plates were further incubated at 37 °C for 4 h. Subsequently, 150 μL of dimethyl sulfoxide (DMSO) was added to each well to dissolve the formazan crystals, and the plate was gently agitated on a water bath shaker at 37 °C for 30 min. The absorbance at 570 nm was measured using a Bio-Rad Model 680 microplate reader.

### 2.9 Western blot

Total cellular proteins were isolated from HK-2 cells following treatment. Protein concentrations were quantified using a BCA assay. Equal amounts of protein were separated by SDS-PAGE and electrophoretically transferred to PVDF membranes. After blocking with 5% skim milk for 1 h, the membranes were incubated with specific primary antibodies and subsequently with appropriate secondary antibodies. Protein bands were visualized using enhanced chemiluminescence (ECL) reagents and captured with an imaging system.

### 2.10 Biodistribution

For *in vivo* fluorescence imaging, HCS-LGP NPs were labeled with FITC. Free FITC and FITC-labeled HCS-LGP NPs were administered intravenously to model rats via the tail vein. At predetermined time points (2, 8, and 24 h), the rats were euthanized, and the kidneys along with other major organs (spleen, liver, lung, and heart) were harvested. The surfaces of the organs were rinsed with 0.9% saline before *ex vivo* fluorescence imaging of FITC was performed using a Maestro *in vivo* imaging system.

### 2.11 Evaluation of *in vivo* treatment effect

The model rats were randomly assigned to three groups (n = 10 per group) and received intravenous injections of 0.9% saline, bulk LGP, or HCS-LGP NPs ([LGP] = 3 mg/kg) every 2 days for a total of seven administrations (Tang et al., 2015). Body weight was recorded every 3 days throughout the study.

After 18 days, blood samples were collected to measure serum levels of urea nitrogen (BUN) and creatinine (SCr) using an automated biochemical analyzer. The rats were then euthanized, and major organs were harvested and fixed overnight in 4% paraformaldehyde at 4 °C. The fixed tissues were embedded in paraffin, sectioned at 4 μm thickness, and stained with hematoxylin and eosin (H&E) for histological examination under a digital microscopy system. Additionally, kidney sections were subjected to Masson’s trichrome staining to evaluate renal damage and collagen deposition. Immunohistochemical (IHC) staining was performed to assess the expression of TGF-β1 and CollagenI in kidney tissues.

### 2.12 Statistical analysis

All data are expressed as mean ± standard deviation. Differences between two comparison groups were tested using Student’s t-test, and one-way analysis of variance (ANOVA) was used to test for significance between multiple groups. *P < 0.05, **P < 0.01, ***P < 0.001, ****P < 0.0001 were considered statistically significant differences.

## 3 Results

### 3.1 The synthesis of HCS polymers

As shown in Figure 1, SDM was conjugated to chitosan (CS) via a succinic acid linker. The structure of HCS was confirmed by FT-IR spectroscopy (Figure 1A). Compared to the spectrum of CS, three new absorption bands appeared at 3,448 cm^−1^, 3,351 cm^−1^, and 3,234 cm^−1^, which were attributed to the N–H stretching vibrations of SDM and the O–H stretching vibration of the succinic acid moiety within HCS. Additionally, two new peaks at 1718 cm^−1^ and 1,560 cm^−1^ were ascribed to two newly formed amide bonds. These results confirm the successful synthesis of the HCS conjugate.

### 3.2 Characterizations of the HCS-LGP NPs

The HCS-LGP NPs and HCS NPs were prepared through self-assembly. As depicted in Figures 1B,C, the HCS NPs showed an average hydrodynamic diameter of 134.9 ± 1.2 nm and a zeta potential of −37.5 ± 3.1 mV. After loading with LGP, the average diameter increased to 178.8 ± 4.4 nm, while the zeta potential changed to −29.9 ± 2.5 mV. The TEM images confirmed the formation of uniform spherical nanoparticles, with individual particles measuring approximately 100–150 nm in diameter (Figure 1D). These results collectively confirm the successful preparation of the drug-loaded HCS-LGP NPs.

To evaluate the drug loading capacity of the HCS-LGP NPs, a standard curve was established using ultraviolet spectrophotometry. The fitted linear regression equation for the calibration curve was as follows. The drug loading percentage of LGP was calculated to be (7.5 ± 0.2)%.

$$y=0.036x+0.0693,R^{2}=0.9963$$

(y: the absorbance intensity of UV-Vis; x: the LGP concentration, µg/mL; the detection limits: 1.0–60.0 μg/mL)

### 3.3 *In vitro* release assay of LGP and HCS-LGP NPs

*In vitro* release profiles of HCS-LGP NPs and free LGP powder were evaluated using a dialysis method, with drug quantification carried out by UV spectrophotometry. As illustrated in Figure 1, free LGP displayed rapid release kinetics: 30% cumulative release was observed at the first sampling point (1 h), and nearly complete release (≈100%) was achieved within 6 h (Figure 1E). In contrast, the HCS-LGP NPs exhibited clear pH-dependent release behavior. The cumulative release rate at pH 5.4 was 44.33% ± 1.15% over 48 h, significantly higher than the rate of 36.33% ± 1.53% at pH 7.4. This enhanced drug release under acidic conditions can likely be attributed to the increased solubility of the HCS matrix at lower pH. Although a slight initial burst release was observed, the nanoparticle system overall enabled markedly sustained drug release compared to free LGP. These findings suggest that HCS-LGP NPs hold promising potential for the development of sustained-release and targeted drug delivery systems.

### 3.4 Cellular uptake

**FIGURE 2:**
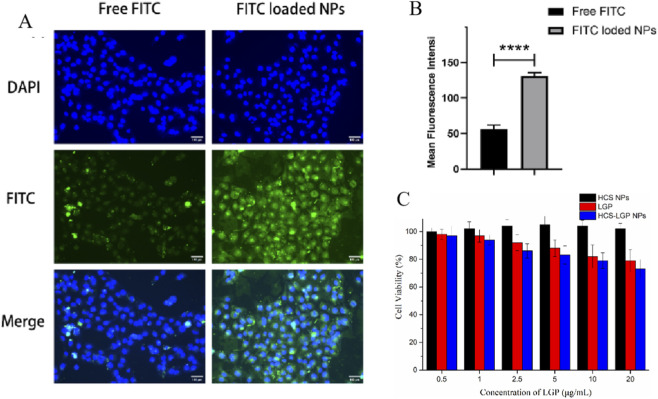
The typical cellular uptake images **(A)** and relative fluorescence intensity **(B)** of FITC and FITC-loaded HCS-LGP NPs to TGF-β1-induced HK-2 cells. Scale bar = 300 μm. **(C)** *In vitro* cytotoxicity of Free LGP, HCS NPs, and HCS-LGP NPs against TGF-β1-induced HK-2 cells at 24 h ****P < 0.0001.

Cellular uptake of free FITC and FITC-labeled HCS-LGP NPs was evaluated in a TGF-β1-induced HK-2 cell fibrosis model. After 5 h of incubation at 37 °C, faint green fluorescence was detected in cells treated with free FITC, whereas the cells treated with the FITC-labeled HCS-LGP NPs showed markedly stronger intracellular fluorescence signals (Figure 2A). The result demonstrated efficient and extensive internalization of HCS-LGP NPs by HK-2 cells. Quantitative fluorescence analysis further confirmed that the cellular uptake efficiency of the HCS-LGP NPs was significantly higher than that of free FITC (Figure 2B). The enhanced drug uptake observed with the chitosan-based nanoparticles may be attributed to chitosan’s structural similarity to glycosaminoglycans, which resemble natural ligands of the megalin receptor, potentially promoting receptor-mediated endocytosis of the nanoparticles.

### 3.5 Cell viability assay

To further explore the potential of HCS-LGP NPs for drug delivery, the MTT assay was employed to evaluate their cytotoxic effects on a TGF-β1-induced HK-2 cell fibrosis model. As shown in Figure 2C, HCS NPs alone showed no cytotoxicity, indicating their good biocompatibility. In contrast, free LGP exhibited significant cytotoxic activity, confirming its therapeutic potential against fibrotic HK-2 cells. Notably, HCS-LGP NPs induced significantly stronger cytotoxicity compared to that of free LGP (p < 0.05). This enhancement is likely attributable to the improved cellular uptake of the nanoparticles, leading to higher intracellular concentrations of LGP. These findings are consistent with and further supported by our cellular uptake results.

### 3.6 *In vivo* renal targeting

To evaluate the renal accumulation of HCS-LGP NPs, FITC was encapsulated into the nanoparticles as a fluorescent probe. Free FITC and FITC-labeled HCS-LGP NPs were administered intravenously to rats via tail vein injection at an equivalent FITC dose. Due to the size of the rats, which precluded whole-body imaging using the *in vivo* imaging system, only *ex vivo* organ imaging was performed at predetermined time points (2 h, 8 h, and 24 h).

**FIGURE 3:**
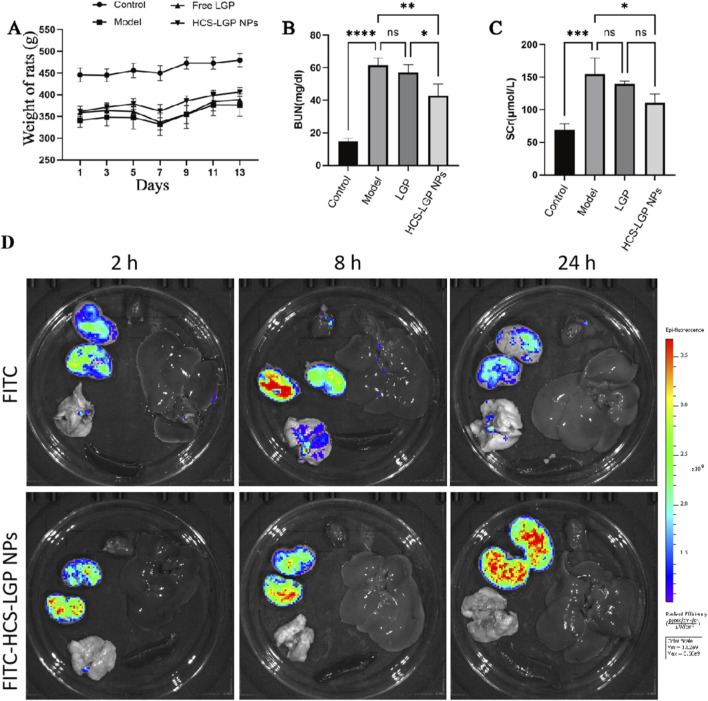
Body weight monitoring and biochemical indices of renal function in rats. Changes in body weight of rats during drug administration **(A)**; serum urea nitrogen level **(B)** and serum creatinine level **(C)**. n = 4,Values are expressed as mean ± SD, *P < 0.05, **P < 0.01, ***P < 0.001, ****P < 0.0001. **(D)** *Ex vivo* fluorescence images of the kidneys, heart, liver, spleen, and lung of the CKD rats injected with free FITC, or FITC-labelled HCS-LGP NPs at different time points (2, 8, 24 h).

As FITC is primarily metabolized by the kidneys, fluorescence intensity was highest in the kidneys compared to other organs (Figure 3A). In the free FITC group, fluorescence peaked at 8 h and subsequently declined, becoming relatively weak by 24 h due to rapid clearance. In contrast, the FITC-labeled HCS-LGP NPs group showed a progressive increase in renal fluorescence from 2 h to 24 h, indicating significantly greater and sustained accumulation in the kidneys compared to other tissues. The prolonged retention further suggested sustained-release characteristics of the nanoparticles.

These results demonstrate that HCS-LGP NPs exhibit renal targeting properties, with specific kidney distribution, extended retention, and controlled release. This targeted delivery suggests the potential for enhanced therapeutic efficacy of LGP and improved biosafety profiles due to reduced off-target exposure.

### 3.7 *In vivo* effect of HCS-LGP NPs

Body weight changes during the administration period are presented in Figure 3B. Rats in the control group maintained normal growth and exhibited significantly higher body weights compared to those in the model, LGP, and the HCS-LGP NPs groups. The reduced weight gain in the model group was attributed to renal fibrotic lesions induced by adenine. Throughout the treatment period, all model rats showed gradual weight recovery. However, rats in the LGP group exhibited a rate of weight gain comparable to that of the model group, indicating limited therapeutic efficacy of free LGP. In contrast, rats treated with the HCS-LGP NPs demonstrated significantly greater weight gain than those in both the model and the LGP groups. These results suggested that HCS-LGP NPs effectively ameliorate the weight loss associated with adenine-induced renal fibrosis.

To evaluate the therapeutic effect of the nanoparticles on renal fibrosis, classic renal function biomarkers—serum creatinine (Scr) and blood urea nitrogen (BUN)—were measured. As shown in Figures 3C,D, both BUN and Scr levels were significantly elevated in the model group compared to the normal control group, indicating impaired renal function. Treatment with free LGP did not lead to significant improvement in these parameters. In contrast, the administration of the HCS-LGP NPs significantly reduced both BUN and Scr levels. These results demonstrated that the HCS-LGP NPs effectively ameliorated the renal impairment of the model rats.

### 3.8 H&E and Masson’s trichrome staining

**FIGURE 4:**
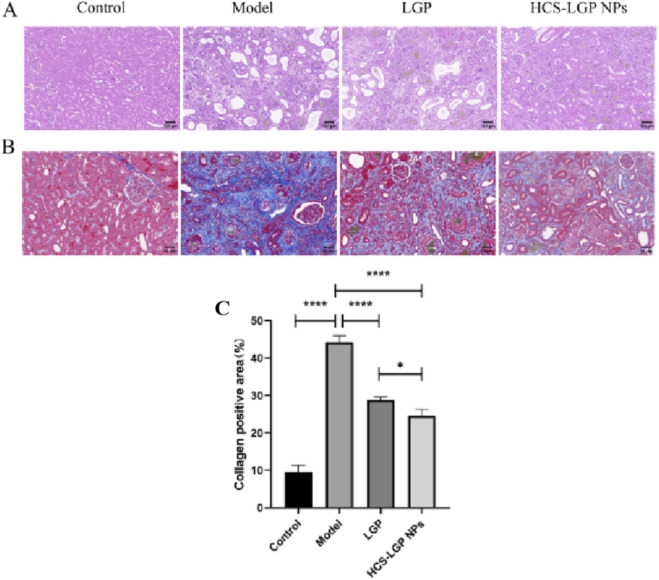
Representative images **(A)** of H&E staining of renal tissues. Bar = 20 μm. Representative images **(B)** of Masson staining of renal tissues and the quantitative analysis of the collagen positive area. Representative images **(C)** Bar = 50 μm. Values are expressed as mean ± SD, *P < 0.05. ****P < 0.0001.

The effects of the nanoparticles on renal fibrosis were assessed using hematoxylin and eosin (H&E) and Masson’s trichrome staining. As shown in Figure 4A, renal tissue sections from rats of the control group displayed no significant histopathological abnormalities. In contrast, renal tissue sections from rats of the model group exhibited disorganized glomerular architecture, pronounced periglomerular fibrosis, and marked tubular dilation. All treatment groups showed considerable improvement in renal injury compared to the model group, with the nanoparticle-treated group exhibiting more substantial recovery than the free-drug group. Masson’s trichrome staining results are presented in Figure 4B. Minimal collagen deposition was observed around glomeruli and tubules in the control group. In comparison, renal tissues from the model group displayed extensive collagen fiber accumulation, indicated by prominent blue staining around these structures. This staining was significantly reduced in the HCS-LGP NPs group. Quantitative analysis further confirmed that the HCS-LGP NPs had a more pronounced anti-fibrotic effect (Figure 4C). These results indicate that encapsulating LGP within nanoparticles enhances its therapeutic efficacy in mitigating renal fibrosis.

### 3.9 Rat kidney tissue immunohistochemistry

**FIGURE 5:**
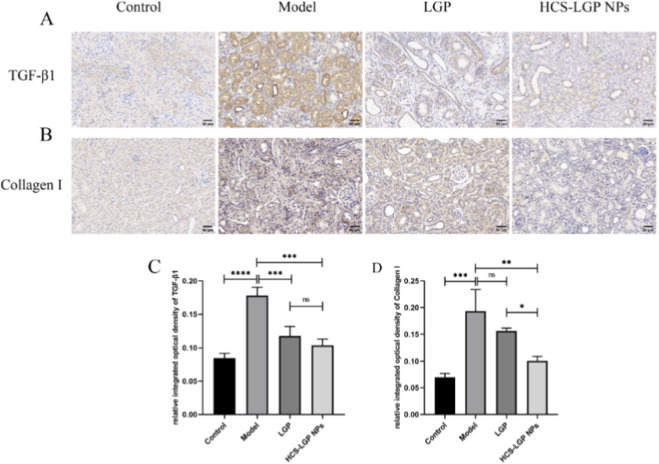
Representative immunohistochemical images of TGF-β1 **(A)** and Collagen I **(B)** in rat kidney tissues. The quantitative analysis of TGF-β1 **(C)** and Collagen I **(D)** in the immunohistochemical images. n = 4, bar = 50 μm.Values are expressed as mean ± SD, *P < 0.05. **P < 0.01, ***P < 0.001, ****P < 0.0001.

In immunohistochemical analyses, the effects of LGP and HCS-LGP NPs on the expression of Collagen I and TGF-β1 in renal tissues were further evaluated (Figure 5). The model group exhibited extensive yellow or brown staining within renal tubules and around glomeruli, indicating elevated TGF-β1 protein expression and Collagen I deposition. Both LGP and HCS-LGP NPs downregulated TGF-β1 protein expression and Collagen I deposition in the kidneys of rats with renal fibrosis. And a more pronounced reduction of the two protein expression could be observed in the HCS-LGP NPs group. These results suggested that both LGP and HCS-LGP NPs could effectively reduce the accumulation of Collagen I and TGF-β1 in fibrotic renal tissues, with HCS-LGP NPs exhibiting superior efficacy.

### 3.10 *In vivo* biocompatibility testing of HCS-LGP NPs

**FIGURE 6:**
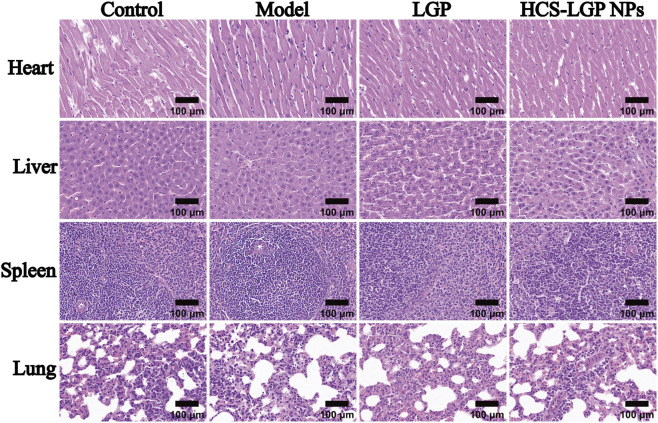
Representative images of H&E staining of the major organs. Bar = 100 μm.

To evaluate the effects of HCS-LGP NPs on major organs in rats, histological analysis via H&E staining was conducted on the heart, liver, spleen, and lungs. As shown in Figure 6, tissue structures in all examined organs remained intact with tightly adherent cells in the Control, Model, and HCS-LGP NPs groups, suggesting that the nanoparticles caused no significant damage to normal tissues *in vivo*. In contrast, the LGP group exhibited signs of liver injury, which may be attributed to the increased metabolic burden on the liver caused by repeated administration of free LGP, resulting in mild hepatic damage. Overall, these findings indicate that the fabricated nanodrug delivery system possesses good biocompatibility and a favorable safety profile.

## 4 Discussion

To enhance the antirenal fibrosis efficacy of LGP, low molecular weight chitosan was selected as a nano-delivery carrier to construct a renal-targeted nanodrug delivery system. Amphiphilic chitosan polymers were successfully synthesized by grafting the hydrophobic compound SDM onto low molecular weight chitosan, as confirmed by infrared spectrophotometry. These amphiphilic polymers self-assembled in aqueous solution to form blank chitosan nanoparticles. LGP was subsequently encapsulated into the hydrophobic core, resulting in the successful preparation of the nanodrug system HCS-LGP NPs, which exhibited a drug loading capacity of (7.5 ± 0.2)%. DLS measurements showed that the average hydrodynamic size and zeta potential of the drug-loaded nanoparticles were 178.8 ± 4.4 nm and −29.9 ± 2.5 mV, respectively. TEM image revealed spherical particles with a uniform size distribution ranging from 100 to 150 nm. Notably, the size (∼180 nm) and moderately negative zeta potential (∼-30 mV) align with established criteria for renal-targeted nanocarriers: this size range minimizes rapid clearance by the mononuclear phagocyte system (MPS) while promoting passive accumulation in fibrotic kidneys via enhanced vascular permeability, and the negative charge further supports prolonged circulation time.

*In vitro* release studies revealed that the HCS-LGP NPs released 36.33% and 44.33% of LGP in phosphate-buffered saline (PBS) at pH 7.4 and 5.4, respectively, over a 48-h period. An initial burst release within the first 10 h promoted rapid achievement of therapeutically effective drug concentrations in renal tissues. This was followed by a sustained release phase from 10 to 48 h, supporting prolonged antifibrotic efficacy.

A series of *in vitro* experiments were conducted to evaluate the anti-renal fibrosis effects of the HCS-LGP NPs. MTT assays indicated that the blank nanocarriers were highly biocompatible, and the HCS-LGP NPs showed significant cytotoxicity to fibrotic HK-2 cells. Cellular uptake studies in HK-2 cells revealed significantly higher internalization of the HCS-LGP NPs compared to free FITC, which is attributed not only to structural similarity between the aminoglycoside moiety of chitosan and the Megalin receptor ligand (promoting receptor-mediated endocytosis) but also to the optimized physicochemical profile of the nanoparticles (size, charge, and amphiphilicity), which facilitates additional non-specific membrane interactions and endocytic pathways. This enhanced cellular uptake suggested that the nanoformulation might improve drug delivery efficiency and strengthen anti-fibrotic effects.

*In vivo* studies indicated that the nanodelivery system accumulated in the kidneys and exhibited sustained drug release. Renal fluorescence imaging showed stronger fluorescence signals in the HCS-LGP NPs group than in the free FITC group, with significantly higher accumulation in the kidneys compared to other organs, demonstrating effective renal targeting. This targeting capability enhanced the anti-fibrotic efficacy of the formulation. In a rat model of renal fibrosis, 14-day treatment with HCS-LGP NPs significantly reduced serum creatinine and blood urea nitrogen levels compared to the model group and the free LGP group. Histopathological examination via H&E and Masson staining indicated that renal fibrosis, glomerular structural disruption, and tubular damage were markedly alleviated in the HCS-LGP NPs group. Immunohistochemical analysis further revealed that the nanoformulation significantly decreased the overexpression and deposition of TGF-β1 and Collagen I in fibrotic renal tissues. H&E staining of major organs (heart, liver, spleen, and lungs) confirmed the good biocompatibility of HCS-LGP NPs. Overall, the targeted nanodelivery system exhibited superior anti-renal fibrosis effects compared to free drug administration.

## 5 Conclusion

In this study, the LGP loaded nanoparticles with uniform size and high stability were successfully synthesized. These nanoparticles, demonstrating accelerated drug release under acidic conditions, were effectively internalized by fibrotic HK-2 cells, and significantly inhibited cell proliferation. Moreover, they markedly suppressed the expression of both TGF-β1 and Collagen I proteins. Animal studies further confirmed the renal-targeting capability of the nanoparticles, which significantly reduced serum levels of urea nitrogen and creatinine without inducing apparent adverse effects.

## Data Availability

The original contributions presented in the study are included in the article/supplementary material, further inquiries can be directed to the corresponding author.
